# Radiation-induced cancer after radiotherapy for non-Hodgkin's lymphoma of the head and neck: a retrospective study

**DOI:** 10.1186/1748-717X-4-21

**Published:** 2009-07-10

**Authors:** Kazuma Toda, Hitoshi Shibuya, Keiji Hayashi, Fumio Ayukawa

**Affiliations:** 1 Department of Radiology, Tokyo Medical and Dental University, 5–45, Yushima 1-chome, Bunkyo-ku, Tokyo 113-8519, Japan; 2 Department of Radiology, Niigata Cancer Center Hospital, Niigata, Japan

## Abstract

**Background:**

survivors of non-Hodgkin's lymphoma (NHL) are well known to be at an increased risk of second malignancies. In this study, we evaluated the incidence and clinical features of head and neck cancer (HNC) occurring after radiotherapy (RT) for NHL.

**Materials and methods:**

We investigated the clinical records of 322 patients who had received RT for early-stage NHL of the head and neck at our institute between 1952 and 2000.

**Results:**

There were 4 patients with a second HNC developing in the irradiated field, consisting of 2 patients with gum cancer, 1 case with tongue cancer and 1 case with maxillary sinus cancer. The pathological diagnosis in all the 4 patients was squamous cell carcinoma (SCC). Two of the patients (one with gum cancer and one with maxillary sinus cancer) died of the second HNC, while the remaining 2 patients are still living at the time of writing after therapy for the second HNC, with neither recurrence of the second tumor nor relapse of the primary tumor. The ratio of the observed to the expected number (O/E ratio) of a second HNC was calculated to be 12.7 (95%CI, 4.07–35.0), and the absolute excess risk (AER) per 10,000 person-years was 13.3. The median interval between the RT and the diagnosis of the second HNC was 17.0 years (range, 8.7 to 22.7 years).

**Conlusion:**

The risk of HNC significantly increased after RT for early-stage NHL. These results suggest that second HNC can be regarded as one of the late complications of RT for NHL of the head and neck.

## Background

Carcinogenesis associated with exposure to radiation is widely known, first reported in the early 20th century, when skin cancer was noted in radiation workers. The risk of carcinogenesis following low-dose radiation exposure was estimated to be 0.05–0.1 Sv based on the results of follow-up of atomic bomb survivors in Japan, however, that associated with exposure to much lower doses, such as that associated with diagnostic X-ray examinations, is debatable [1,2]. Exposure to therapeutic doses of radiation has also been shown to be associated with an increased risk of a second cancer, although the precise risk remains unknown. For selected cancers with a high cure rate, the benefits of treatment need to be weighed against the potential risk of treatment-related second malignancy.

Progress of therapeutic modalities in recent decades have considerably improved the prognosis of malignant lymphoma, on the other hand, development of therapy-related second cancer as a late complication of treatment has became obvious [3-6]. As compared with the case in HL, RT still occupies a more important position in the treatment of NHL, especially early-stage NHL. Although the head and neck area is one of the most frequent sites of NHL, the risk of a second HNC after RT for NHL still remains unclear. We investigated the incidence and clinical features of a second HNC occurring after RT for early-stage NHL.

## Materials and methods

We conducted a retrospective review of a total of 322 patients who received RT with/without chemotherapy as initial therapy for early-stage NHL (stage I or stage II) of the head and neck at our institute between 1952 and 2000 [7]. The patient parameters investigated were the sex, age at the time of RT, the chemotherapy regimen employed, the clinical stage and location of the lymphoma, the irradiated field, the dose and type of RT, and the cause of death.

For the patients in whom a second HNC developed in the irradiated field after RT, we investigated the site and pathological diagnosis, the interval from the time of RT to the diagnosis of the second HNC, and the clinical course of the second cancer. We calculated the expected numbers of second cancers by using the person-years method [8,9]. We used the age-, sex-, and calendar year-specific cancer incidence rates in the general population of Japan [10]. O/E ratio was then calculated with the 95%CI from the Poisson distribution. These results were statistically analyzed by the SPSS for Windows (SPSS Inc. Chicago, Illinois).

## Results

The patient characteristics are listed in Table 1. In all, 96 patients had NHL lesions in the Waldeyer's ring. Extranodal lesions were seen in 124 patients. The most frequent site of NHL was the oral cavity (n = 48). Neoadjuvant and/or adjuvant chemotherapy was administered in 144 patients (44.7%), and the most frequently administered regimen was cyclophosphamide, doxorubicin, vincristine+ prednisolone (CHOP) or a CHOP-like regimen (n = 88).

**Table 1:** Characteristics of all the patients (n = 322)

	n	%
Sex		
Male	191	59.3
Female	131	40.7
Age at the time of RT (median, 53 years{range,4 – 91})		
<60 years	200	62.1
≧ 60 years	122	37.9
Stage		
I	200	62.1
II	122	37.9
Chemotherapy		
+	144	44.7
-	178	55.3

Follow-up duration after RT		
Average(range)	8.6 years(0 – 35.1)	

Abbreviations RT: radiotherapy		

RT was administered with high-voltage X-rays from a linear accelerator in 150 patients, with γ-rays from Co-60 in 89 patients, with orthovoltage X-rays in 55 patients, with either high-voltage X-rays or γ-rays plus electrons in 15 patients, with electrons alone in 9 patients, with high-voltage X-rays plus γ-rays in 2 patients, with γ-rays plus orthovoltage X-rays in 1 patient, and orthovoltage X-rays plus brachytherapy in 1 patient. RT was administered with conventional RT techniques, therefore 1 field, 2 opposed fields and a combination of them were mostly used.

The median total dose of RT was 40.8 Gy (range, 5.5–78 Gy), and the dose per fraction was 1.5–3 Gy (2 Gy in most cases). The total radiation dose was unknown in the patient who received low-dose-rate intracavitary brachytherapy in addition to orthovoltage X-rays for NHL of the tonsil. The total dose employed was 5.5–19.8 Gy in 9 patients (2.8%), 21–30 Gy in 64 patients (19.9%), 30.8–40 Gy in 87 patients (27.1%), 40.8–50 Gy in 143 patients (44.5%), 50.6–60 Gy in 16 patients (5.0%), and over 60 Gy in 2 patients (0.6%).

**Figure 1:**
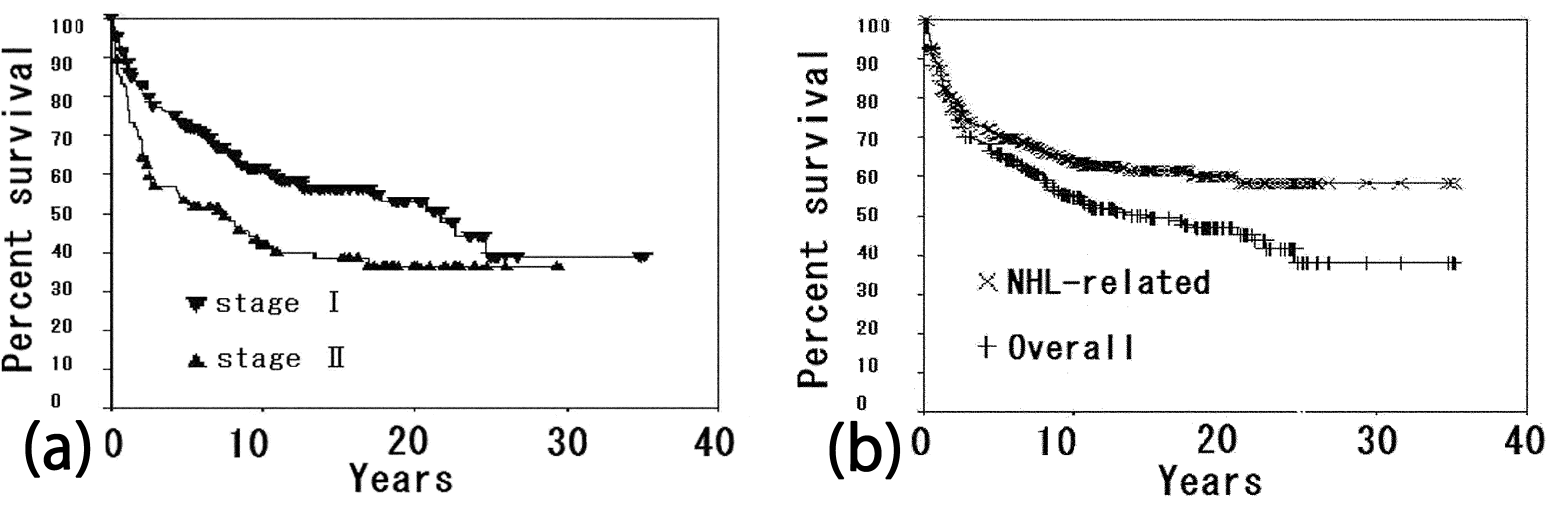
**(a) Overall survival and lymphoma-specific survival rates in NHL patients**. (b) Overall survival of NHL patients by stage.

The overall 2-, 5- and 10-year survival rates of the patients calculated by the Kaplan-Meier method were 77.6%, 65.5% and 54.7%, respectively. The median survival time was 14.9 years (95%CI, 8.2–21.5 years). The lymphoma-related 2-, 5- and 10-year survival rates were 80.1%, 70.4% and 63.7%, respectively. There was a significant difference in the overall survival rate between NHL patients with clinical stage I and those with clinical stage II (p < 0.05) (Fig. 1).

**Figure 2:**
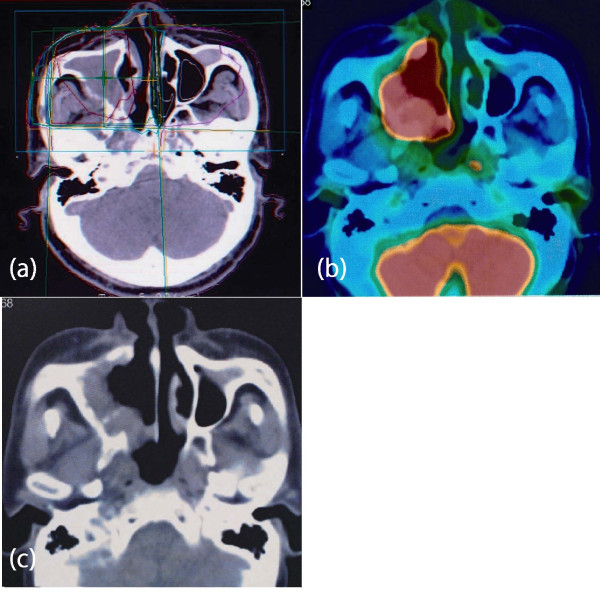
**(a) Dose distribution of RT for NHL of the maxillary sinus**. (b) and (c) PET-CT showing second SCC infiltrating the bone.

Of the patients, 19 (5.9%) developed a second malignancy, which was metachronous in 16 cases and synchronous in 3 cases (table 2). In 4 patients, the second HNC occurred in the irradiated field. The clinical outlines of these 4 patients are shown in [table S1; Additional file 1]. Two of the 4 patients had also received chemotherapy (3 cycles of CHOP). The pathological diagnosis of the second HNC in all the 4 cases (2 cases of cancer of the gum, 1 case of tongue cancer, and 1 case of maxillary sinus cancer) was SCC (fig. 2). The median interval after the RT to the development of the second cancer was 13.9 years (range, 8.7 to 22.7 years). Two of the patients (1 with gum cancer and 1 with maxillary sinus cancer) died of the second cancer. The remaining 2 patients are still living at the time of writing, with neither recurrence of the second HNC nor relapse of the primary NHL, or indeed any severe complications during the follow-up. The patient with gum cancer is still living, 3.3 years after surgery for SCC of the right upper gum, and the patient with tongue cancer is also still living, 8.9 years after RT for SCC of the tongue. The latter case received 90.5 Gy as brachytherapy for tongue cancer by Au-198 grain implantation.

**Table 2:** Characteristics of the second tumor(n = 19)

Type of second tumor	n	O/Eratio	95%CI	AER*
Synchronous	3			
Esophagus	1			
Stomach	1			
Cervix	1			
				
Metachronous	16	0.8	0.47–1.33	-14.6
Head and neck				
In irradiated field	4	12.7	4.07–35.0	13.3
Out of irradiated field	1			
Esophagus	2	3.24	0.56–13.1	4.95
Stomach	2	0.39	0.07–1.55	-11.4
Colon	3	1.55	0.40–4.93	3.80
Breast	1			
Gallbladder	1			
Soft-tissue sarcoma(buttocks)	1			
Myeloma(thoracic vertebra)	1			

* Absolute excess risk per 10,000 person-years

During the 2776 person-years (PYs) of observation, the expected number of a second HNC in the general population was 0.31, so that the O/E ratio was 12.7 (95%CI, 4.07–35.0, p < 0.01). The absolute excess risk (AER) of a second HNC per 10,000 PYs was 13.3. When the analysis was limited to the 192 patients who could be followed up for over 5 years, the expected number was 0.28 during 2544 PYs, the O/E ratio was 14.1 (95%CI, 4.5–38.7, p < 0.01), and the AER was 14.6. Furthermore, the O/E ratio was 12.0 (95%CI, 2.1–48.4, p < 0.01) during 1600 PYs in the 178 patients who did not receive chemotherapy, and 13.5 (95%CI, 2.3–54.5, p < 0.01) during 1176 PYs in the144 patients who received chemotherapy.

Of the 19 patients with a second cancer, 2 cases of second cancer arose near the previous radiation field: one of laryngeal cancer developing 14 years after RT for NHL of the nasal cavity, and one of esophageal cancer developing 16 years after RT and chemotherapy for NHL of the oral cavity and neck.

## Discussion

Some definitions of radiation-induced malignancy have been proposed. We removed the 2 second cancers (one each of laryngeal cancer and esophageal cancer) which arose near the radiation field from the analysis of radiation-induced cancer according to the criteria that Sakai et al. proposed, even though these cases might well have had a relation to scattered radiation [11]. Cahan et al. reported their criteria for the diagnosis of radiation-induced osteosarcoma in the middle of last century [12]. According to their criteria, the primary lesion for which RT was administered must be a benign disease. In the early part of the last century, RT was widely used for benign diseases such as tuberculous lymphadenitis, skin diseases, thyroid diseases and spondylitis, however, at present, RT is mainly used to treat malignancies. The limitation of the prior disease treated by RT to a benign disease might thus be impractical. Sakai et al. argued the criteria for the diagnosis of radiation-induced cancer, except leukemia, and their results suggested that the reliability of the diagnosis of radiation-induced cancer depends on the pathological diagnosis, the organ of origin, the follow-up duration after RT (over 5 years) and on whether the lesion is located in the irradiated field [11]. These criteria were based on the criteria of double primary cancer proposed by Warren et al. [13].

A limitation of our study is that our study population was small. A long latency period of radiation-induced malignancies except leukemia would make it difficult to analyze these malignancies [11,12,14]. Therefore, a large number of patients who have been under observation for a long time after RT would be necessary to correctly assess a radiation-induced cancer. Tward et al reported that the O/Eratio of a second HNC among 77823 NHL patients was 1.28 (95%CI, 1.12–1.46) [5]. British group reported that the O/Eratio of a second HNC among 5519 HL patients was 2.8(95%CI, 1.1–5.8) and that among 2456 NHL patients was 2.6(95%CI, 0.8–6.0) [4,6]. These studies showed that RT alone did not significantly relate to a second HNC, although the relationship between the details of RT and a site of second malignancies was not considered. In this study, O/Eratio of a second HNC was higher than those previously reported and significantly increased even among the patients who received RT alone. The possibility that those large-scale studies underestimated carcinogenicity of RT because of the lack of consideration for the details of RT could not be ruled out, although our study population was smaller than that in previous studies.

Chemotherapy for NHL is held to be associated with a certain risk of carcinogenesis, especially of leukemia, lung cancer, bladder cancer and colorectal cancer [4]. No cases of second leukemia and second lung cancer were observed in this study. The lack may be affected by a strong relationship between these second malignancies and chemotherapy. Chemotherapy occupied a relatively lower place in the therapy for NHL than in that for HL, at least especially in the earlier decades. For example, only about 27% of all the patients received CHOP that is now standard regimen for B-cell NHL in combination with rituximab and CHOP-like regimens in this study. The increased risk of second malignancies in the synergy of radiation and chemotherapy is also known, although how the synergy affected induction of second HNC was unknown.

The risk of certain malignancies is significantly associated with smoking, habitual alcohol consumption, immunosuppressive conditions, and some genetic disorders. It would appear that the higher risk of a second cancer in patients with HNC remains even after they stop smoking [15]. Moertel et al. described multicentric cancer development associated with carcinogenic stimulation of large areas of tissues [16,17]. Slaughter et al. proposed "field cancerization" in oral SCC [18]. These reports underscore the difficulty of distinguishing radiation-induced malignancy from not only recurrence of the first malignancy, but also from multicentric primary tumors in the head and neck area [19]. We did not have sufficient data about the smoking, alcohol drinking habit, and genetic disorders of all the patients. We do know, though, that only 1 of the 4 patients with a second HNC had a smoking history, and that none of them engaged in habitual alcohol consumption.

A dose-response relationship is known in the development of leukemia in experimental animals. The incidence of leukemia was reported to increase with the radiation dose in the dose range between 3 and 10 Gy [20]. The explanation for the decrease in the incidence at higher doses is that the number of surviving cells decreases at these doses. A similar relationship was suggested between sarcoma induction and the radiation dose employed, with the maximum dose levels for malignant transformation and decreased cell survival being higher than those for leukemia [21]. This is held to be one of the reasons why sarcomas are likely to be induced in heavily irradiated tissues. A relationship between initial RT doses and second head and neck malignancies was unknown in this study. The RT doses employed in our study were relatively lower than those used for other solid tumors, and likely to be higher than those used for NHL in today. To be concrete, about half of all the patients including 4 second HNC patients received 40 Gy and over. No other pathological diagnosis than SCC was seen in second head and neck malignancies and this result was similar to previous studies [22,23]. In contrast, Sale et al. reported 13 second malignancies of the head and neck after RT, with the most frequent histological diagnosis being sarcoma, followed in frequency by SCC [24]. And Patel et al. reported 10 patients of radiation-induced sarcoma of the head and neck, and malignant fibrous histiocytoma was the commonest pathological diagnosis (4 patients) in their patient series [25]. The difference of the pathological diagnosis among these studies might be related to the difference of RT doses, nevertheless a correct relationship between initial RT doses and second head and neck malignancies is unclear because of a small number of these malignancies.

Equipment and techniques mainly used for RT in today are likely to differ from those used for our patients. About half of our patients were treated with a linear accelerator which is now standard RT equipment, and almost all the patients were treated with conventional RT techniques. However, how advance of radiation techniques affects second malignancies is held to be debatable. Intensity modulated radiation therapy (IMRT) which is one of the advanced RT techniques is concerned to increase the risk of a second cancer compared with three-dimensional conformal radiotherapy (3D-CRT) [26,27]. The change from 3D-CRT to IMRT involves a bigger volume of normal tissue irradiated by lower doses as a result of the increase of fields, of monitor units and of scattered radiation. In contrast, Ruben at al. argued that the risk of radiation-induced cancer did not significantly differ between IMRT and 3D-CRT concerning the body in totality, and the risk of second cancer was regarded to be influenced by RT equipment [28]. At least, it must be inappropriate to simply apply our results to NHL patients treated with modern RT equipment and techniques.

It is debatable whether the prognosis of radiation-induced malignancy might differ from that of spontaneously occurring tumors. Previous studies on radiation-induced sarcoma suggested a poor prognosis of these patients and also the beneficial effects of surgery for these tumors [21,25,29-33]. In addition, the poor prognosis of radiation-induced sarcoma of the head and neck might be related to the difficulty in complete resection of these tumors due to post-radiation changes [25]. It was held that surgery should be conducted prior to RT in the treatment of radiation-induced cancer, because of the lowered tolerance of the tissues to re-radiation and the oxygen effect of the second tumor [19]. McHugh et al. compared the characteristics of radiation-induced craniofacial osteosarcoma with those of the corresponding primary tumors, and proposed that the poorer prognosis of radiation-induced osteosarcoma was related to the higher expression of adverse prognostic markers, such as p53, TP53 mutations, ezrin expression, and the higher proliferative activity [34]. In contrast, there are some reports of laryngeal and pharyngeal cancer after radiation for thyrotoxicosis and tuberculous lymphadenitis being successfully treated by radiation from a linear accelerator [35,36]. The choice of the therapeutic modality for radiation-induced cancer is affected not only by the nature of the tumor, but also by several patient factors, mainly the extent of the existing tissue damage. Because of the small number of cases, we could not estimate the prognosis of second HNC after RT for NHL. However, we assumed that patients with a second cancer after RT for NHL would have a little advantage over those with a radiation-induced cancer after the treatment of other solid tumors. Because relatively lower radiation doses given for lymphomas than those for solid tumors would lead to a lower extent of damage of the surrounding tissue, patients with a second cancer after RT for NHL might show better tolerance to treatment for the second tumor. Therefore, an early detection of second HNC may aid in a better choice of a therapeutic modality. Of course, an irradiated area is ought to be under careful observation. In addition, observations of NHL patients ordinarily include systemic follow-up that may encourage a detection of second primary cancer even distant from an irradiated area.

## Conclusion

The risk of HNC significantly increased after RT for early-stage NHL, although a precise relationship between RT and second head and neck malignancies remains unclear because of a small number of cases. Anyway, we propose to regard second HNC as one of the late complications after RT for NHL of head and neck.

## Competing interests

The authors declare that they have no competing interests.

## Authors' contributions

KT and HS designed/conducted analysis and wrote the manuscript. KH and FA assisted in the acquisition and analysis of data. All authors have read and approved the final manuscript.

## Supplementary Material

Additional file 1 **Table S1**. Characteristics of the radiation-induced head and neck cancer patients (n = 4). Click here for file
