# Supplementary material for: Radiation-induced cancer after radiotherapy for non-Hodgkin's lymphoma of the head and neck: a retrospective study
Source: Radiat Oncol. 2009 Jul 10;4:21. doi: 10.1186/1748-717X-4-21 (PMC2717106; doi:10.1186/1748-717X-4-21)
Supplement: Additional file 1 — Table S1. Characteristics of the radiation-induced head and neck cancer patients (n = 4). [file 1748-717X-4-21-S1.doc]

| Table S1. Characteristics of the radiation-induced head and neck cancer patients (n = 4) | Second head and neck cancer | Outcome | Follow-up term(y)* | 8.9 | 3.6 | 3.3 | 0.8 | Abbreviations RT : radiotherapy,  * Time since treatment for second cancer. |
| --- | --- | --- | --- | --- | --- | --- | --- | --- |
|  |  |  | Status | Alive | Dead | Alive | Dead |  |
|  |  | Therapy | | Brachy-  Therapy | Surgery and RT | Surgery | Chemo-  therapy |  |
|  |  | Site | | Tongue | Gum | Gum | Maxillary sinus |  |
|  |  | Age / Time since RT(y) | | 68/22.7 | 54/11.3 | 61/16.6 | 69/8.7 |  |
|  |  |  |  |  |  |  |  |  |
|  | Primary non-Hodgkin’s lymphoma | Therapy | | 60 Gy | 40 Gy | 45 Gy and 3 cycles of CHOP | 45 Gy and 3 cycles of CHOP |  |
|  |  | Stage | | Ⅱ | Ⅱ | Ⅰ | Ⅰ |  |
|  |  | Age/Sex | | 45/F | 42/M | 45/M | 61/M |  |
